# Influence of low- and high-elevation plant genomes on the regulation of autumn cold acclimation in *Abies sachalinensis*

**DOI:** 10.3389/fpls.2015.00890

**Published:** 2015-10-21

**Authors:** Wataru Ishizuka, Kiyomi Ono, Toshihiko Hara, Susumu Goto

**Affiliations:** ^1^Forestry Research Institute, Hokkaido Research OrganizationBibai, Japan; ^2^Institute of Low Temperature Science, Hokkaido UniversitySapporo, Japan; ^3^Graduate School of Agricultural and Life Sciences, The University of TokyoTokyo, Japan

**Keywords:** cold acclimation, interpopulation variation, elavational cline, phenology, *Abies sachalinensis*, modeling, paternity analysis

## Abstract

Boreal coniferous species with wide geographic distributions show substantial variation in autumn cold acclimation among populations. To determine how this variation is inherited across generations, we conducted a progeny test and examined the development of cold hardening in open-pollinated second-generation (F_2_) progeny of *Abies sachalinensis*. The F_1_ parents had different genetic backgrounds resulting from reciprocal interpopulational crosses between low-elevation (L) and high-elevation (H) populations: L × L, L × H, H × L, and H × H. Paternity analysis of the F_2_ progeny using molecular genetic markers showed that 91.3% of the fathers were located in surrounding stands of the F_1_ planting site (i.e., not in the F_1_ test population). The remaining fathers were assigned to F_1_ parents of the L × L cross-type. This indicates that the high-elevation genome in the F_1_ parents was not inherited by the F_2_ population via pollen flow. The timing of autumn cold acclimation in the F_2_ progeny depended on the cross-type of the F_1_ mother. The progeny of H × H mothers showed less damage in freezing tests than the progeny of other cross-types. Statistical modeling supported a linear effect of genome origin. In the best model, variation in freezing damage was explained by the proportion of maternally inherited high-elevation genome. These results suggest that autumn cold acclimation was partly explained by the additive effect of the responsible maternal genome. Thus, the offspring that inherited a greater proportion of the high-elevation genome developed cold hardiness earlier. Genome-based variation in the regulation of autumn cold acclimation matched the local climatic conditions, which may be a key factor in elevation-dependent adaptation.

## Introduction

Seasonal growth cycles are well described for plant taxa distributed in boreal, sub-boreal, and temperate climates. In particular, for evergreen coniferous species, cold acclimation often occurs before temperatures drop below freezing (Aitken and Hannerz, 2001; Chuine, 2010). During this acclimation, evergreen plants shift their physiological condition from an active growth phase to a hardening phase that involves some degree of freezing tolerance. This physiological shift is responsible for a series of mechanical and biochemical changes in the cell: for example, intracellular accumulation of compatible osmolytes such as soluble sugars, accumulation of polypeptides and/or proteins, and the vesiculation of vacuoles (Fujikawa et al., 2006). The timing of cold acclimation affects fitness. A long duration of the hardy phase may help plants avoid autumn and spring freezing damage. However, it can also limit annual photosynthetic activity and growth. Therefore, because of a trade-off between growth and risk of freezing damage, the timing of phenological events can be a key driver for adaptation to cold climates. During their long evolutionary histories, conifers have acquired an adaptive schedule of cold acclimation to survive under their local environmental conditions, using changes in daylength and/or temperature as regulatory signals (Kozlowski and Pallardy, 2002). Population differences in the acclimation schedule can be observed within species that inhabit wide geographic distributions spanning diverse climatic regions. Interpopulational genetic variation in phenological traits is associated with climatic differences (Rehfeldt, 1989; Oleksyn et al., 1998). Furthermore, interpopulational genetic variation for many boreal conifers is greater during autumn cold acclimation than during spring dehardening (Aitken and Hannerz, 2001; Howe et al., 2003). It is a consistent trend that populations from high latitudes and elevations exhibit earlier development of cold hardening than those from low latitudes or elevations (Rehfeldt, 1989; Skrøppa and Magnussen, 1993; Oleksyn et al., 1998; Savolainen et al., 2004; Notivol et al., 2007; Mimura and Aitken, 2010).

Common garden trials and reciprocal transplantation experiments are powerful tools for detecting adaptive variation in plants and the drivers of natural selection. A previous study using transplanted materials of the sub-boreal conifer *Abies sachalinensis* Mast. (Sakhalin fir) has revealed an adaptive cline in the timing of autumn cold acclimation along an elevational gradient (Ishizuka et al., 2015). In that study, earlier development of autumn cold acclimation in trees derived from high-elevation populations than in those from low-elevation populations was detected at all transplantation sites. Modeling analysis was then conducted to examine the environmental conditions responsible for the detected physiological variation. The results have shown that the genetic variation in response to the temperature change might be an important driver of elevation-dependent adaptation (Ishizuka et al., 2015). However, the mechanisms by which the genome generates quantitative adaptive differences are not clearly understood. Quantitative traits resulting as a consequence of genome inheritance and genome effects are complex (Sykes et al., 2006; Cané-Retamales et al., 2011). Furthermore, some studies have reported epigenetic phenomena which have regulated phenological traits (Johnsen et al., 2005; Kvaalen and Johnsen, 2008). Perennial plants have a “memory” of the climate they experienced during embryogenesis. These epigenetic signals may be transmitted through several mechanisms, including cytosine methylation and histone modification, and may enhance adaptation at a population level (Johnsen et al., 2005; Kvaalen and Johnsen, 2008). Controlled crosses using mother plants that have been exposed to the same environmental conditions can be used to test the effects of genome inheritance on the regulation of the autumn cold acclimation. However, previous ecological studies have not extensively evaluated the complex effects of genome inheritance on the development of cold acclimation in trees.

**FIGURE 1:**
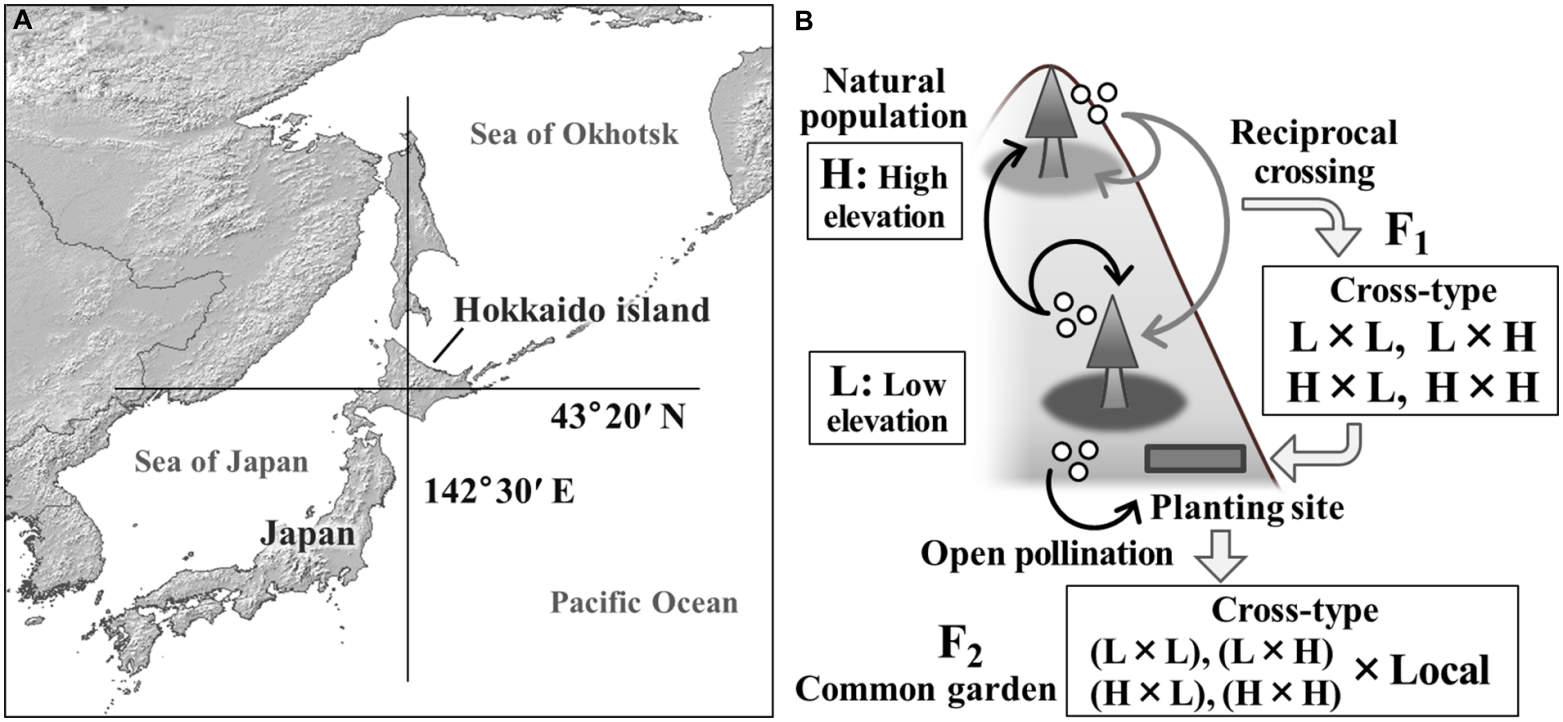
**A map of the study site **(A)** and the experimental design using F_2_ progeny of interpopulational crosses of *Abies sachalinensis***(B)**.** Two natural populations used for crossing were located where the latitudinal and longitudinal lines cross in panel **(A)**. A planting site of the F_1_ population and a common garden trial for the F_2_ progeny were established in the same area. The F_1_ population was produced using reciprocal crossing between low-elevation (L) and high-elevation (H) populations in 1979. Open-pollinated seeds were collected from the F_1_ mother trees in 2009. The resulting F_2_ progeny had various genetic backgrounds resulting from the maternal cross-type.

We investigated the genetic basis of variation in the regulation of autumn cold acclimation using Sakhalin fir. This conifer plays an important role in the timber production in northern Japan. It is a dominant climax species in natural sub-boreal forests of the Far East, thus forming an important component of forest ecosystems (Kato, 1952). The geographic range of this species extends from eastern Siberia to Hokkaido, the northernmost island of the Japanese archipelago (**Figure 1A**). The elevational distribution extends from 100 to 1600 m a.s.l. This species survived in a glacial refugium in Hokkaido, and had become a dominant species by the time of the last glacial maximum, 20000 years ago (Igarashi, 1996; Tsumura and Suyama, 1998). Interpopulational variation in several traits has been observed within this elevational range, including variation in growth and survival (Goto et al., 2011; Ishizuka and Goto, 2012), resistance to biological stresses such as disease or rats (Kurahashi and Hamaya, 1981; Saho et al., 1994), and cold hardiness (Eiga and Sakai, 1984). In our recent study, we reported that clinal variation in the timing of cold acclimation appears to result from adaptation to the local climate at elevations ranging from 230 to 1200 m (Ishizuka et al., 2015).

In the present study, we used open pollination to obtain second-generation (F_2_) progeny for testing the genetic basis of variation in phenological traits. The maternal population (F_1_) was produced by reciprocal crosses between two distinct ecotypes: a population inhabiting low elevations and a population inhabiting high elevations. The genetic background of the maternal F_1_ trees varied among cross-types (low × low, hybrids between elevations, high × high), and the trees were planted at a single site in order to ensure their exposure to the same environment. Thus, we could evaluate the effects of genome inheritance by examining the traits of the F_2_ population. Using the F_2_ progeny, we conducted (1) paternity analysis based on microsatellite markers and (2) statistical modeling of freezing damage. Our objectives were to (1) measure variation in the timing of autumn cold acclimation of the F_2_ progeny and (2) establish whether this variation could be explained by the genomic background inherited from the low-elevation or high-elevation populations.

## Materials and Methods

### F_1_ Planting Site

We conducted this study in the University of Tokyo Hokkaido Forest (UTHF), located in central Hokkaido, northern Japan (43°20′N, 142°30′E; **Figure 1A**). In the UTHF, natural sub-boreal forests cover about 150 km^2^ of the south-western slope of Mt. Dairoku (1459 m a.s.l.), with an elevational gradient of more than 1200 m. With increasing elevation, dramatic changes occur in soil type, snow depth, and forest vegetation, including changes in the prevalence of the bamboo species, *Sasa senanensis* and *Stigmella kurilensis* (Kato, 1952; Toyooka et al., 1983; Nakata et al., 1994). However, the primary factor changing with elevation is air temperature. Based on monitoring data recorded hourly from 2009 to 2010 at 230 m, the annual and winter (October–March) mean temperatures were 6.53°C and -1.78°C, respectively. Temperatures at 1100 m were 1.75°C and -6.43°C, respectively.

Sakhalin fir, *A. sachalinensis*, grows from the lowland (200 m) to the high-elevation zone (1200 m) in this area. In 1979, natural populations at 400–530 m and 1100–1200 m were selected as low-elevation (L) and high-elevation (H) populations, respectively, and artificial reciprocal crossing was performed by Kurahashi (Kurahashi, 1995; Goto et al., 2011). Within each population, five and three adult trees were selected as mother (seed parents) and father trees (pollen donors), respectively (Kurahashi and Hamaya, 1981). Pollen from the three father trees in each population was pooled and used for the crosses. The generated progeny were categorized into four cross-types (female × male): L × L, L × H, H × L, and H × H (**Figure 1B**). We refer to these progeny as the F_1_ population. Seedlings of the F_1_ population were grown in an outdoor nursery at a lowland site of the UTHF. In 1986, the F_1_ individuals were transplanted to the lowland planting site (220 m; **Figure 1B**). At the planting site, the progeny were arrayed in grids at a spacing of 1.2 m, assigning one block (2 columns × 10 rows) to each cross-treatment. Unreplicated blocks were used to avoid contamination among treatments (Kurahashi, 1995). In total, 440 F_1_ seedlings were transplanted. Thus, the F_1_ populations had varying genetic backgrounds but were exposed to the same environmental conditions. Further information about the traits measured on the F_1_ trees (survival, height, diameter at breast height, leaf nitrogen content, and leaf area per weight) were provided by Goto et al. (2011).

### Seed Collection from the F_1_ Generation

In 2009, 23 years after planting, 353 F_1_ trees were alive and some of them were in a reproductive stage. Our field surveys during the flowering and seed-maturing seasons showed that 22 trees flowered that year at the F_1_ planting site (**Table 1**). We collected open-pollinated seeds from the flowering trees on September 6th and 9th, 2009. We selected 13 F_1_ mother trees that produced a sufficient number of progeny (>20 cones) to use in this study (**Table 1**; see the Supplemental Table for ID-information of the mother trees). The progeny (F_2_ population) were grouped into four types according to the maternal cross category: L × L, L × H, H × L, and H × H (**Figure 1B**). Collected seeds were stored at -3°C until use.

**Table 1:** Number of F_1_ trees of *Abies sachalinensis* that were living, flowering, or used to collect open-pollinated seeds in 2009.

Cross-type	Living	Flowering	Seed collection
L × L	144	7	3
L × H	62	4	3
H × L	59	4	3
H × H	88	7	4
**Total**	353	22	13

*Four types of F_1_ trees were planted at 220 m a.s.l. These trees were originated from controlled reciprocal crosses between low-elevation (L) and high-elevation (H) populations.*

### F_2_ Common Garden Trial

We conducted a common garden trial of the F_2_ progeny. Before the trial, viable seeds were selected from all collected seeds using soft X-ray photography (Softex, Kanagawa, Japan). In the spring of 2010, the viable seeds were subjected to a seed stratification treatment and were sown into the UTHF nursery (230 m a.s.l.; **Figure 1A**). One seed was placed in each 4-cm grid of a 1.0 m × 1.2 m block to maintain the identity of all germinated seedlings. The four blocks were separated by buffer spaces. To avoid edge effects for the F_2_ progeny, control seeds were also sown at the edge of the rows and columns. In total, 2112 seeds were sown in this trial (four blocks × 22 rows × 24 columns). The seedlings were raised until the end of the second growing season. As shown in **Table 2**, the cross-type differences of the mother of the F_2_ progeny were reflected in some functional traits, such as the germination rates and 2-year heights.

**Table 2:** Germination rates and 2-year heights of F_2_ progeny in a common garden trial at 230 m a.s.l.

Cross-type of mother	Germination rate (%)	2-year height (mm)
L × L	82.7 (16.1)	80.1 (21.6)
L × H	76.3 (12.8)	72.6 (20.0)
H × L	81.3 (13.2)	77.5 (17.6)
H × H	73.8 (7.8)	63.6 (18.7)
**Total**	79.3 (14.0)	74.7 (20.9)

*The F_2_ progeny were categorized into four cross-types, according to the cross-types of the F_1_ mother trees. Standard deviations are shown in parentheses.*

### Genotyping using SSR Markers

To estimate the genome composition of the F_2_ progeny, we performed molecular genetic analysis using nuclear microsatellite (nSSR) and chloroplast microsatellite (cpSSR) markers. In the present study, we used four nSSRs: As08, As16, As32 (Lian et al., 2007), and NFH 15 (Hansen et al., 2005), and three cpSSRs: pt30204, pt71936 (Vendramin et al., 1996), and pt30249 (Liepelt et al., 2001).

Samples selected for SSR genotyping consisted of the 22 flowering F_1_ trees described in **Table 1** plus 80 of their F_2_ progeny. These 80 F_2_ progeny were derived from seeds collected in 2009, and were composed of 16 progeny from each of five F_1_ trees selected across the cross-types (**Table 3**). These seedlings were germinated in an indoor growth chamber.

**Table 3:** Genetic diversity statistics of the F_1_ trees that flowered in 2009 and their open-pollinated F_2_ progeny.

Population	Mother	*N*	*A*	*H*o	*H*_E_	*F*_IS_
F_1_ (flowered in 2009)		22	10.3	0.864	0.838	-0.033
F_2_	As-F_1_-C034	16	9.0	0.875	0.735	-0.199
	As-F_1_-C042	16	7.5	0.906	0.750	-0.216
	As-F_1_-C148	16	8.8	0.906	0.757	-0.198
	As-F_1_-C168	16	7.8	0.906	0.749	-0.212
	As-F_1_-C061	16	8.8	0.906	0.771	-0.175
	**Total**	80	8.4	0.900	0.752	-0.200

*N, number of individuals sampled; *A*, average number of alleles; *H*_*O*_, observed heterozygosity; *H*_*E*_, expected heterozygosity; *F*_*IS*_, fixation index.*

For the DNA extractions, foliage was collected from the 22 candidate F_1_ trees and the 80 F_2_ progeny. DNA was extracted using the DNeasy Plant Mini Kit (Qiagen, Ltd., Crawley, UK), and the PCR reactions were performed using the Multiplex PCR Kit (Qiagen, Ltd., Crawley, UK) following the protocol of Lian et al. (2007). The PCR products were sequenced using an Applied Biosystems 3130xl Genetic Analyzer (Life Technologies, Carlsbad, CA, USA). Genotyping of all samples was performed based on the length of each sequenced fragment using Peak Scanner ver. 1.0 software from Applied Biosystems. Genetic diversity statistics were calculated by GenAlEx ver. 6.5 (Peakall and Smouse, 2012).

### Paternity Analysis

We considered the 22 flowering F_1_ trees as candidate pollen parents of the F_2_ progeny because no other trees were flowering at the F_1_ planting site. However, trees outside of the F_1_ population may have also served as pollen parents because the F_1_ planting site was surrounded by mature artificial forests. The origin of these artificial forests was a local lowland forest (200–300 m) that was different from the forests used to create the F_1_ population.

For paternity analysis, we first inferred paternity of the F_2_ progeny using four nSSRs. Using CERVUS 3.0 (Kalinowski et al., 2007), we compared the nSSRs genotypes of the 80 F_2_ progeny to their known seed parents (mothers) and 22 candidate F_1_ pollen parents (fathers). The most likely father was estimated using maximum-likelihood assignment from the possible allele combinations (genotypes) of the parents. When none of the potential F_1_ donors were assigned as fathers, we considered pollen contamination and genotyping errors as possible causes. Genotyping errors can lead to inflated estimates of outside pollen flow (Slavov et al., 2005). We set the genotyping error to 1% with an 80% confidence level for the 10000 cycle-simulations for the paternity assignment.

After assigning fathers using CERVUS, we performed a simple exclusion procedure using three cpSSRs. The F_2_ progeny and the assumed F_1_ pollen parents were required to have matching cpSSR haplotypes (*Abies* cpSSRs are paternally inherited; Vendramin and Ziegenhagen, 1997). We assumed that CERVUS assigned the correct pollen parent when the genotypes of the F_2_ progeny and assigned pollen parent matched for all three cpSSR markers (considering genotyping errors). When a complete match was not found, we assumed the pollen came from outside of the F_1_ planting site.

Combining the results from CERVUS and the exclusion procedure, we estimated the proportion of selfed seeds, seeds sired by the F_1_ population, and seeds sired by fathers outside the studied F_1_ population. We also estimated the composition of the F_1_ trees among four cross-types, based on the genotyping results.

### Autumn Freezing Test

We conducted freezing tests on the F_2_ progeny three times during the autumn season using the method of Eiga and Sakai (1984). The potted seedlings were placed outdoors just before the test. Three freezing tests were conducted at approximately 2-week intervals: on 12 and 26 October, and 11 November (referred to as Tests 1, 2, and 3). Each freezing test was performed at the Institute of Low Temperature Science, Hokkaido University. The potted seedlings were placed in a dark chamber kept at 5°C. After an overnight incubation, the temperature was lowered at a rate of 2°C per hour until the target temperature was reached. The target temperature was maintained for 4 h, and then increased at a rate of 2°C per hour to 5°C. For Test 1, the target temperature was -15°C. For Tests 2 and 3, the target temperatures were -15 and -30°C. The seedlings were kept in the growth chamber for 14 days with a 12-h photoperiod (the photosynthetic photon flux density was 100 μmol m^-2^ s^-1^ during the day). We measured freezing damage using the visual scoring method of Lindgren and Hallgren (1993). Freezing damage was scored using six classes of needle discoloration (brown needles) as follows: 0 (no damage), 1 (1–20% of needles were discolored), 2 (21–40% discolored), 3 (41–60% discolored), 4 (61–80% discolored), and 5 (81–100% discolored). For each temperature at each time point, we used 10 F_2_ seedlings from each F_1_ mother tree. For some maternal trees with fewer seedlings, we used seven seedlings. In total, 605 seedlings were used in the freezing test (121 seedlings × 5 test conditions).

### Data Analysis

Because no freezing damage was observed in the -15°C treatment in Tests 2 and 3, we excluded these data from subsequent analysis. Therefore, three test conditions were defined: T_1_ involved freezing at -15°C in Test 1, T_2_ involved freezing at -30°C in Test 2, and T_3_ involved freezing at -30°C in Test 3. For each condition, we used a nested ANOVA to study the effect of maternal cross-type and the effect of mother trees on freezing damage in the F_2_ progeny, using the following model:

(Model 1) $$Y_{ijk}=\mu +C_{i}+M_{j}(C_{i})+E_{ijk},$$

where *Y*_ijk_ is the freezing damage score of the *k*-th progeny (*k*; 1-10) of the *j-*th mother tree (*j*; 1-4) in the *i*-th cross-type (*i*; 1-4), μ is the general mean, *C*_i_ is the effect of the *i*-th cross-type, *M*_j_ (*C*_i_) is the effect of the *j*-th mother tree nested in the *i*-th cross-type, and *E*_ijk_ is the residual error.

We also evaluated the effect of low-elevation and high-elevation plant genomes on freezing damage using the following statistical model that includes all test conditions. We assumed that freezing damage in the F_2_ progeny is affected by the genetic inheritance, test conditions, and mother tree. Therefore, we constructed the full model with all assumed effects, as follows:

(Model 2–5) $$Y_{ijkl}\;=\mu +C_{i}+T_{l}+C_{ij}\times T_{l}+M_{j}(C_{i})+M_{j}(C_{i})\times T_{l}+E_{ijkl},$$

where *Y*_ijkl_ is the freezing damage score of the *k*-th progeny of the *j-*th mother tree in the *i*-th cross-type under the *l*-th test condition (*l*; 1-3), μ, *C*_i_ and *M*_j_ (*C*_i_) are the same as in Model 1, and *T*_l_ is the effect of the *l*-th test condition. In this full model, we regard the model element *C* as representing the effect of the plant genome (genetic effect). Then, in the analysis, *C, T*, and their interaction (*C* × *T*) are treated as fixed effects, whereas *M* (*C*) and its interaction with *T* are treated as random effects. Because damage is an ordinal variable (Ishizuka et al., 2015), we used an ordered probit mixed model (Lee, 1992). Specifically, we used the cumulative link mixed model (CLMM) function in the “ordinal” package of R 3.1.2 (R Development Core Team, 2014). CLMM uses a maximum likelihood approach with the Laplace approximation and adaptive Gauss-Hermite quadrature (see Christensen, 2014).

We then assessed which types of variables were appropriate for the effect of the plant genome (*C*) to describe the freezing damage score. Therefore, we used the following four types of the genetic effects as *C* in Models 2–5:

Model 2: Linear effect (0 for L × L; 0.25 for L × H and H × L; 0.5 for H × H)
Model 3: Categorical effect (L × L; L × H; H × L; H × H)
Model 4: Categorical interaction effect (“NoHyb” for L × L and H × H; “Hyb_LH_” for L × H; “Hyb_HL_” for H × L)
Model 5: Linear effect + interaction effect (0 for L × L; 0.25 + α for L × H; 0.25 - α for H × L; 0.5 for H × H).

In Model 2, the proportion of high-elevation genome inherited from the maternal parent was used. Numeric values indicating the linear effect of the maternal genome origin were assigned to *C*. The highest value was assigned to the H × H cross-type, whereas the lowest value, 0, was assigned to the L × L cross-type. Model 2 should show a good fit if freezing damage was closely related to the amount of high-elevation genome inherited from the maternal trees (i.e., quantitative trait). In Model 3, *C* was set to one of four categorical values indicating the cross-type of the maternal trees. Model 3 should fit well if the maternal origin is important, but the proportion of high-elevation genome is not. Model 4 included a genome interaction effect. As described above, *C* was set to one of three categorical values: NoHyb, Hyb_LH_, or Hyb_HL_. In Model 5, we included both the cross-type linear effect and genome interaction effect. *C* was partitioned into the combination of the variables used in Models 2 and 4.

We used a model selection procedure to exclude the variables that did not improve the model fit. The Akaike Information Criterion (AIC) was used to consider goodness-of-fit and number of parameters (Johnson and Omland, 2004). Within each model (Models 2–5), a backward stepwise procedure from the full model was performed to determine the most effective variable sets. Then, we compared the AIC values among all candidate models and selected the model with the lowest AIC value as the best model for explaining freezing damage in the F_2_ progeny. All statistical analyses in this study were conducted using R 3.1.2 (R Development Core Team, 2014).

## Results

### Paternity Analysis

All seven SSR markers were polymorphic. For the nSSR markers, the average number of alleles per locus (*N*) was 15.0, ranging from 7 for As08 to 21 for As16 and NFH15. For the cpSSR markers, the numbers of haplotypes of Pt30141, Pt30204, and Pt71936 were 15, 13, and 7, respectively. The genotypes of the 22 F_1_ candidate pollen parents differed from each other (see Supplemental Table). Genetic diversity statistics for the four nSSR markers are summarized for the F_1_ and F_2_ materials in **Table 3**. The genetic characteristics of the F_2_ progeny were consistent among their maternal groups. The average number of alleles in the F_2_ progeny was smaller than that in the F_1_ trees, which was caused by maternal sharing (13 out of 353 F_1_ trees; **Table 1**). In contrast, their observed heterozygosity (*H*_O_) was higher in the F_2_ progeny than in the F_1_ trees, resulting in a negative fixation index (*F*_IS_).

Paternity analysis using four nSSR markers showed that 12 F_2_ progeny had a father from the candidate F_1_ pollen parent group. By adding the results of cpSSR analysis, seven F_2_ progeny out of 12 were not excluded. For these seven F_2_ progeny, the cross-type of all of the assigned F_1_ fathers was L × L. There were no progeny with genotypes composed solely of maternal alleles, excluding the possibility of selfing. Moreover, most of the F_2_ progeny had novel alleles in comparison with the candidate F_1_ pollen donors. Allowing for genotyping errors, the proportions of selfing, mating between F_1_ trees, and mating with outside trees were 0, 8.7 (7 samples), and 91.3% (73 samples), respectively.

### Autumn Freezing Test and Model Selection

**FIGURE 2:**
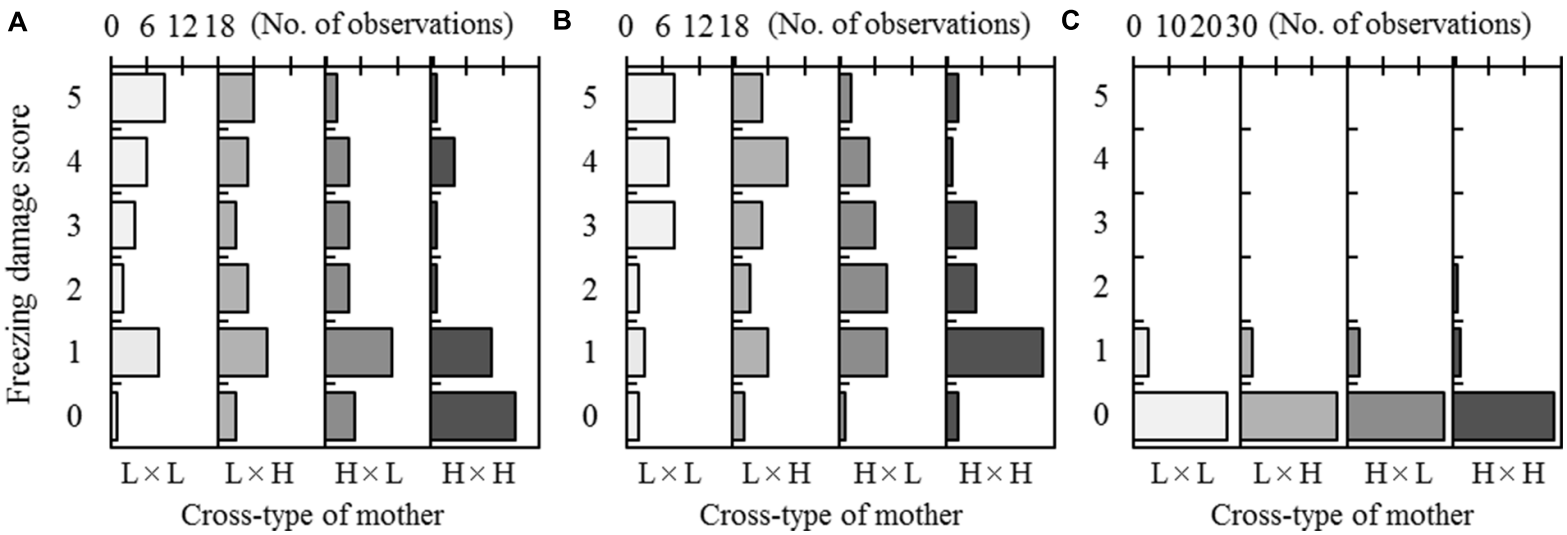
**Freezing damage of F_2_ progeny after a freezing test at -15°C in Test 1 **(A)**, -30°C in Test 2 **(B)**, or -30°C in Test 3 **(C)**.** Three freezing tests were conducted in autumn at a 2-week interval. The freezing damage scores range from 5 (complete needle discoloration) to 0 (no discoloration). Observed frequencies were shown by arraying the cross-type of each mother (on the lower x-axis). The maximum scale of the number of observations (on the top x-axis) was set to 18 for each of the four cross-types in Tests 1 and 2, and set to 30 for each of the four cross-types in Test 3.

Freezing damage scores in the F_2_ progeny ranged from 0 to 5 for treatment T_1_ (-15°C in Test 1) and treatment T_2_ (-30°C in Test 2; **Figure 2**). In T_1_, the most frequent damage scores for the progeny belonging to the L × L and H × H cross-types were 5 (30.0%) and 0 (45.2%), respectively. For the hybrid cross-types (L × H and H × L), a score of 1 was most common (26.7% for L × H and 36.7% for H × L). In T_2_, the most frequent damage score for the H × H cross-type was 1 (51.6%). In contrast, high damage scores (4 and 5) were often observed for the L × L, L × H, and H × L cross-types. In T_3_ (-30°C in Test 3), most progeny showed no damage, regardless of the cross-type (**Figure 2C**).

The ANOVA indicated a significant difference among mother trees within cross-types [*M* (*C*)] for treatment T_1_ (**Table 4**). For treatment T_2_, a significant difference was detected among maternal cross-types (*C*), but not among mother trees [*M* (*C*)]. For treatment T_3_, however, statistical analysis was not possible due to limited variation in freezing damage score, as shown in **Figure 2**.

**Table 4:** ANOVA of freezing damage in the F_2_ progeny for each test condition, including degrees of freedom (DF), mean square (MS) and *F*-value (*F*).

		T_1_		T_2_		T_3_	
Source	*DF*	*MS*	*F*	*MS*	*F*	*MS*	*F*
*C*	3	21.47	3.30	13.46	**4.54**^∗^	0	**–**
*M (C)*	9	6.50	**2.78**^∗∗^	2.97	1.48	0	**–**
Error	108	2.34		2.00		0	

*C, cross-type; *M* (*C*), mother tree nested in the cross-type; T_1_, -15°C in Test 1; T_2_, -30°C in Test 2; T_3_, -30°C in Test 3. ^∗^*P* < 0.05, ^∗∗^*P* < 0.01.*

Model selection for each of the four candidate models (Models 2–5) indicated that the interaction terms *C* × *T* and *M* (*C*) × *T* should be excluded from Models 2, 3, and 5 (**Table 5**). In Model 4, the *C* effect was also excluded. This indicates the effect of the mother trees assumed in Model 4 (i.e., non-hybrid vs. hybrid) was not useful for predicting freezing damage in the F_2_ progeny. A model comparison based on AIC values revealed that Model 2, which included the cross-type linear effect, was the best model (**Table 5**). The effects included in this model were *C, T*, and *M* (*C*). The estimated coefficients of these variables are shown in **Table 6**. A significant negative effect was observed for *C*, indicating that the freezing damage tended to be lower in the progeny that inherited a higher proportion of the high-elevation genome.

**Table 5:** Candidate model components and Akaike Information Criterion (AIC) values after model selection.

Candidate models	Model components					AIC
	*C*	*T*	*C* × *T*	*M* (*C*)	*M (C)* × *T*	
Model 2	+ (Linear)	+	-	+	-	**813.23**
Model 3	+ (Categorical)	+	-	+	-	815.10
Model 4	- (Categorical interaction)	+	-	+	-	822.90
Model 5	+ (Linear with categorical interaction)	+	-	+	-	814.99

*For each model, variables were retained (represented by +) or excluded (-) by a stepwise procedure. The lowest AIC value, shown in bold, indicates the best model. In Models 2–5, different types of effects were used for model component *C*.*

*C, T, C × *T*: fixed effect of plant genome, the test condition, and their interaction, respectively.*

*M (*C*), *M* (*C*) × *T*: random effect of the mother tree nested in the cross-type and its interaction with test condition.*

**Table 6:** Estimated parameters for Model 2, the best model among all candidate models (Models 2–5) for predicting freezing damage in the F_2_ progeny (standard errors are in parentheses).

Model 2		Coefficient	*p*-value
Fixed effect	*C* (Linear)	-2.440 (0.580)	<0.001
	*T* (T1)	0	-
	(T_2_)	0.336 (0.136)	0.014
	(T_3_)	-8.277 (101.7)	0.935
Random effect	*M (C)* (13 categories)	≈0 (0.206)	

*The negative effect of plant genome (*C*) indicates that the progeny with a higher proportion of the high-elevation genome tend to show less freezing damage.*

## Discussion

Freezing damage differed among seedlings of *A. sachalinensis* early in the study period, but was barely observed in the final test conducted in November (**Figure 2**). This demonstrates the development of freezing tolerance over time. In addition, freezing damage differed among maternal cross-types of the F_2_ progeny, even though the mother trees were grown in the same environment (**Figure 2**). Genetic variation in autumn phenology was relatively clear. Seedlings that inherited a greater proportion of the high-elevation genome displayed earlier cold acclimation. We used visual scoring, but alternative quantitative evaluations (e.g., by chlorophyll fluorescence or electrolytic leakage) are also used as indicators of cold hardiness (Lindgren and Hallgren, 1993; Ehlert and Hincha, 2008). Although quantitative evaluation is a powerful tool, the accuracy and efficiency of these alternative measures may be compromised if the plants have small leaves. In the present study, most seedlings developed thin needles, making a quantitative measurement difficult. In contrast, discoloration occurred evenly, making differences in freezing damage clear and comparable among seedlings. In addition, there is a strong correlation (*r* = 0.762) between our scoring method and chlorophyll fluorescence (evaluated by the *F*_v_/*F*_m_ value) in *A. sachalinensis* (Ishizuka et al., 2015). Thus, our evaluation method is sufficient for detecting phenotypic variation associated with cold acclimation in this species.

In studies of other boreal conifers, genetic variation in autumn phenology and associations with the climate of origin indicated adaptation to the local climate (Rehfeldt, 1989; Skrøppa and Magnussen, 1993; Oleksyn et al., 1998; Savolainen et al., 2004; Notivol et al., 2007; Mimura and Aitken, 2010). In our previous study using reciprocally transplanted materials of *A. sachalinensis*, we found clinal variation in the timing of autumn cold acclimation along an elevational gradient (Ishizuka et al., 2015). Here, we further evaluated the effects of genetic background of the trees using molecular markers and statistical models to improve our understanding of the local adaptation and the evolution of autumn cold acclimation.

### Paternity Analysis

The combined use of nSSR and cpSSR markers made it possible to assign pollen parents to *A. sachalinensis* seedlings. We assigned 22 candidate F_1_ pollen parents to the F_2_ progeny with consideration for genotyping error and assignment confidence (Slavov et al., 2005). We assumed that cryptic gene flow had little effect because the genetic origin of the F_1_ trees was different from that of the artificial forests surrounding the F_1_ planting site. Therefore, when the paternity analysis found no possible fathers among the candidates, we assumed that the pollen parent was outside of the F_1_ planting site, and presumably derived from the surrounding mature plantations.

The small paternal contribution from the F_1_ trees (8.7%) indicated a high level of pollen flow from outside pollen sources (91.3%). High levels of pollen contamination have been reported to be a severe problem in seed orchard management (Wheeler and Jech, 1992). When a seed orchard is young, the proportion of outside pollen flow tends to be extremely high. For example, Ozawa et al. (2009) performed paternity analysis of collected seeds in a young *Pinus densiflora* seed orchard, and detected 82% outside pollen flow. The results of that study suggested that the amount of pollen brought to the female strobili from the male strobili within the seed orchard was markedly lower than that of immigrant pollen brought from surrounding mature forests. In the present study, only 6.2% of the F_1_ parents were flowering due to their young age (29 years old) and small size (**Table 1**). As suggested by Ozawa et al. (2009), the amount of pollen from the F_1_ planting site must be lower than that from the surrounding mature plantations. Therefore, the results obtained by paternity analysis are reasonable.

If the timing of pollen shed from surrounding plantations and the receptivity of female strobili of the F_1_ trees are mismatched, outside pollination will occur rarely. Indeed, Sasaki (1983) reported that the flowering phenology of *A. sachalinensis* at the plantation forest at 1100 m was delayed by approximately 2 weeks compared with that at 530 m. If the difference in flowering phenology is genetically controlled, the timing of female flowering of F_1_ trees derived from a crossing among high-elevation parents may differ from that of male flowering of local (low-elevation) trees. However, the flowering phenology of the F_1_ trees which were planted at the same environment overlapped, as described by Goto et al. (2011). These results suggest that environmental factors, rather than ontogenetic control, strongly affected the flowering phenology of *A. sachalinensis* (Sasaki, 1983; Goto et al., 2011). Therefore, it is not surprising that outside pollen flow was predominant at our F_1_ planting site.

Evidence of frequent outcrossing was also seen in the genetic parameters obtained using nSSRs. Observed heterozygosity (*H*_O_) increases and the fixation index (*F*_IS_) decreases when mating occurs between local and non-local populations (Hamrick and Godt, 1996). In our study, *H*_O_ of the F_2_ was higher than that of natural stands (Lian et al., 2008; **Table 2**). A large decrease in *F*_IS_ was also detected between the F_1_ and F_2_ generations, although there were only small differences within the F_2_ (**Table 2**). The negative *F*_IS_ in F_2_ indicated that the chances of mating between an F_1_ mother and its relatives were low. These results support our conclusions concerning pollen flow from outside the F_1_ planting site.

We concluded that only a few F_1_ fathers contributed to the F_2_ progeny because of the high levels of pollen flow from the surrounding mature plantations. Moreover, the F_1_ trees that were assigned as fathers (seven progeny) were all derived from the L × L cross-type. This indicates that the high-elevation plant genome from the F_1_ population was not inherited by the F_2_ population to any great degree.

### Autumn Freezing Test and Model Selection

Our modeling analysis revealed that the regulation of autumn cold acclimation could be well explained by the linear effect of the genome origin. The model using the expected proportion of high-elevation genome as a fixed effect was selected as the best model (**Table 5**). In this model, variation in autumn cold acclimation was explained by variation in the maternal genome composition. The F_2_ progeny that inherited a greater proportion of the high-elevation genome showed earlier cold acclimation.

Because of epigenetic phenomena, such as maternal effects and ‘after’ effects, the prediction of phenotypic values for quantitative traits can be a complex process (Howe et al., 2003; Johnsen et al., 2005; Kvaalen and Johnsen, 2008; Cané-Retamales et al., 2011). In some cases, the performance of the progeny is subjected to a significant epigenetic effect (Johnsen et al., 2005; Kvaalen and Johnsen, 2008). An experiment with *Picea abies* revealed that autumn phenological traits (date of bud set) was related to the daylength and/or thermal conditions during fertilization and seed maturation (Johnsen et al., 2005). Moreover, the performance of the progeny may be influenced by phenomena related to epistasis (Sykes et al., 2006). Sykes et al. (2006) used the crossbred progeny derived from a specific combination of crosses in *P. taeda*, and successfully measured the effects of genomic interactions in the chemical contents of woody materials. In the present study, however, the model analysis indicated that epigenetic phenomena and the cross-type-dependent maternal effect were not likely to have played a major role (**Table 5**). In comparison with models considering such effects, the model including only the effect of genome origin showed the best fit. This result could be partly explained by the fact that all maternal trees were exposed to the same environmental conditions. Moreover, in the absence of a specific cross-type effect, it would be reasonable to expect that the autumn cold acclimation of *A. sachalinensis* would show a linear pattern based on the proportion of low-elevation and high-elevation plant genomes.

This interpopulational regulation of autumn phenology may be one of the factors driving the local adaptation of conifers (Aitken and Hannerz, 2001; Howe et al., 2003). For *A. sachalinensis*, clinal variation in the timing of autumn cold acclimation along an elevational gradient is one of the elevation-dependent adaptive traits. Each population adapts to the local climate, with a trade-off between the avoidance of freezing damage and extension of the growth period (Ishizuka et al., 2015). The genome-based control of phenological traits may be the mechanism responsible for the elevation-dependent adaptation of this species.

The genetic control of ecologically relevant traits is important in forest management. Potential genetic changes in future generations must be taken into consideration, particularly considering projections of rapid global warming (Jump and Penuelas, 2005; Aitken et al., 2008; Ishizuka and Goto, 2012). If a target species shows strong genetic variation that is larger than the phenotypic plasticity, it may be important to redistribute the genes that confer optimal performance in the changing climate (Aitken et al., 2008). For *A. sachalinensis*, it may be difficult to track rapid climate change because of the mismatch of locally adapted autumn phenology. However, the presence of adaptive genes and their potential for climatic adaptation have not been sufficiently examined. Future studies may be needed to evaluate this subject in detail by applying powerful molecular techniques, such as the analysis of quantitative trait loci and/or genome scanning.

## Conflict of Interest Statement

The authors declare that the research was conducted in the absence of any commercial or financial relationships that could be construed as a potential conflict of interest.
