# Supplementary material for: Influence of low- and high-elevation plant genomes on the regulation of autumn cold acclimation in Abies sachalinensis
Source: Front Plant Sci. 2015 Oct 21;6:890. doi: 10.3389/fpls.2015.00890 (PMC4617160; doi:10.3389/fpls.2015.00890)
Supplement: Supplementary file 1 [file Table_1.DOCX]

***Supplementary Material***

**Influence of low- and high-elevation plant genomes on the regulation of autumn cold acclimation in *Abies sachalinensis***

**Wataru Ishizuka^1*^, Kiyomi Ono^2^, Toshihiko Hara^2^, Susumu Goto^3^**

^1^ Forestry Research Institute, Hokkaido Research Organization, Hokkaido, Japan

^2^Institute of Low Temperature Science, Hokkaido University, Hokkaido, Japan

^3^Graduate School of Agricultural and Life Sciences, the University of Tokyo, Tokyo, Japan

*** Correspondence:** Wataru Ishizuka, Forestry Research Institute, Hokkaido Research Organization, Hokkaido, Japan.

wataru.ishi@gmail.com

**Supplementary Data**

Supplemental Table: Results of SSRs analysis for 22 F_1_ trees flowered in 2009 and 80 F_2_ progeny.

Supplemental Table: Results of SSRs analysis for 22 F_1_ trees flowered in 2009 and 80 F_2_ progeny.

The listed values are fragment length of PCR products using each marker. First eight values are the results of four nuclear SSRs, and later three are those of chloroplast SSRs. For the F_2_ progeny, we added information of their mother trees.

| Sample type | Experiment Lot | ID | Mother | As32  (1) | As32  (2) | NFH15  (1) | NFH15  (2) | As08  (1) | As08  (2) | As16  (1) | As16  (2) | Pt30204 | Pt30141 | Pt71936 |
| --- | --- | --- | --- | --- | --- | --- | --- | --- | --- | --- | --- | --- | --- | --- |
| F1 donor | As517 | As-F1-C019 | - | 80 | 93 | 124 | 128 | 174 | 180 | 205 | 228 | 83 | 145 | 152 |
| F1 donor | As521 | As-F1-C023 | - | 82 | 82 | 110 | 124 | 174 | 180 | 224 | 228 | 83 | 145 | 152 |
| F1 donor | As534 | As-F1-C061 | - | 80 | 84 | 110 | 128 | 174 | 175 | 203 | 205 | 86 | 147 | 151 |
| F1 donor | As595 | As-F1-C141 | - | 82 | 93 | 126 | 141 | 176 | 180 | 209 | 222 | 87 | 148 | 153 |
| F1 donor | As608 | As-F1-C168 | - | 77 | 93 | 139 | 147 | 176 | 178 | 219 | 222 | 90 | 151 | 151 |
| F1 donor | As630 | As-F1-C202 | - | 82 | 93 | 110 | 124 | 174 | 180 | 224 | 224 | 83 | 145 | 152 |
| F1 donor | As649 | As-F1-C236 | - | 59 | 77 | 116 | 139 | 176 | 180 | 217 | 226 | 90 | 151 | 154 |
| F1 donor | As655 | As-F1-C242 | - | 59 | 82 | 110 | 139 | 176 | 180 | 203 | 217 | 90 | 151 | 154 |
| F1 donor | As661 | As-F1-C248 | - | 59 | 80 | 120 | 139 | 174 | 176 | 222 | 226 | 90 | 151 | 151 |
| F1 donor | As704 | As-F1-C304 | - | 77 | 93 | 128 | 131 | 180 | 180 | 206 | 226 | 83 | 145 | 152 |
| F1 donor | As709 | As-F1-C309 | - | 59 | 77 | 135 | 139 | 176 | 180 | 217 | 226 | 83 | 145 | 152 |
| F1 donor | As715 | As-F1-C327 | - | 80 | 82 | 110 | 128 | 174 | 178 | 205 | 210 | 86 | 147 | 151 |
| F1 donor | As735 | As-F1-C347 | - | 77 | 77 | 112 | 128 | 176 | 178 | 206 | 238 | 83 | 145 | 152 |
| F1 donor | As745 | As-F1-C357 | - | 77 | 77 | 110 | 128 | 178 | 180 | 206 | 238 | 83 | 145 | 152 |
| F1 donor | As750 | As-F1-C362 | - | 77 | 77 | 110 | 135 | 178 | 180 | 206 | 210 | 83 | 145 | 152 |
| F1 donor | As753 | As-F1-C034 | - | 82 | 82 | 128 | 141 | 176 | 179 | 205 | 228 | 88 | 149 | 152 |
| F1 donor | As754 | As-F1-C035 | - | 82 | 91 | 108 | 128 | 174 | 178 | 221 | 228 | 87 | 148 | 155 |
| F1 donor | As755 | As-F1-C037 | - | 82 | 91 | 124 | 128 | 174 | 178 | 228 | 228 | 89 | 150 | 151 |
| F1 donor | As756 | As-F1-C042 | - | 59 | 80 | 110 | 124 | 179 | 179 | 209 | 210 | 86 | 147 | 152 |
| F1 donor | As758 | As-F1-C148 | - | 84 | 93 | 131 | 147 | 176 | 180 | 209 | 222 | 93 | 154 | 154 |
| F1 donor | As759 | As-F1-C264 | - | 93 | 93 | 128 | 128 | 176 | 180 | 205 | 217 | 83 | 145 | 152 |
| F1 donor | As760 | As-F1-C273 | - | 82 | 93 | 128 | 128 | 176 | 178 | 204 | 216 | 83 | 144 | 151 |
| F2 progeny | As_sdl01 | As-F2-01 | As-F1-C034 | 82 | 91 | 124 | 141 | 179 | 180 | 205 | 209 | 88 | 149 | 152 |
| F2 progeny | As_sdl02 | As-F2-02 | As-F1-C034 | 77 | 82 | 116 | 141 | 174 | 179 | 205 | 209 | 93 | 154 | 152 |
| F2 progeny | As_sdl03 | As-F2-03 | As-F1-C034 | 77 | 82 | 126 | 128 | 176 | 178 | 217 | 228 | 87 | 148 | 153 |
| F2 progeny | As_sdl04 | As-F2-04 | As-F1-C034 | 82 | 93 | 128 | 131 | 176 | 179 | 205 | 222 | 93 | 154 | 154 |
| F2 progeny | As_sdl05 | As-F2-05 | As-F1-C034 | 82 | 82 | 128 | 151 | 176 | 179 | 217 | 228 | 78 | 139 | 155 |
| F2 progeny | As_sdl06 | As-F2-06 | As-F1-C034 | 82 | 84 | 126 | 128 | 178 | 179 | 205 | 210 | 88 | 149 | 157 |
| F2 progeny | As_sdl07 | As-F2-07 | As-F1-C034 | 59 | 82 | 128 | 128 | 174 | 179 | 205 | 217 | 85 | 146 | 152 |
| F2 progeny | As_sdl08 | As-F2-08 | As-F1-C034 | 82 | 84 | 130 | 141 | 174 | 179 | 228 | 232 | 86 | 147 | 155 |
| F2 progeny | As_sdl09 | As-F2-09 | As-F1-C034 | 82 | 82 | 108 | 141 | 178 | 179 | 205 | 228 | 87 | 148 | 155 |
| F2 progeny | As_sdl10 | As-F2-10 | As-F1-C034 | 80 | 82 | 114 | 128 | 176 | 178 | 205 | 205 | 91 | 152 | 153 |
| F2 progeny | As_sdl11 | As-F2-11 | As-F1-C034 | 82 | 82 | 120 | 141 | 176 | 179 | 209 | 228 | 86 | 147 | 150 |
| F2 progeny | As_sdl12 | As-F2-12 | As-F1-C034 | 82 | 82 | 130 | 141 | 176 | 176 | 205 | 205 | 88 | 149 | 152 |
| F2 progeny | As_sdl13 | As-F2-13 | As-F1-C034 | 82 | 84 | 118 | 141 | 176 | 180 | 204 | 205 | 82 | 143 | 152 |
| F2 progeny | As_sdl14 | As-F2-14 | As-F1-C034 | 59 | 82 | 126 | 141 | 178 | 179 | 228 | 234 | 87 | 148 | 153 |
| F2 progeny | As_sdl15 | As-F2-15 | As-F1-C034 | 59 | 82 | 122 | 128 | 174 | 176 | 205 | 228 | 86 | 147 | 152 |
| F2 progeny | As_sdl16 | As-F2-16 | As-F1-C034 | 59 | 82 | 110 | 141 | 174 | 176 | 206 | 228 | 87 | 148 | 152 |
| F2 progeny | As_sdl17 | As-F2-17 | As-F1-C042 | 80 | 91 | 110 | 128 | 174 | 179 | 210 | 221 | 87 | 148 | 155 |
| F2 progeny | As_sdl18 | As-F2-18 | As-F1-C042 | 59 | 82 | 110 | 128 | 174 | 179 | 209 | 226 | 88 | 149 | 152 |
| F2 progeny | As_sdl19 | As-F2-19 | As-F1-C042 | 80 | 91 | 108 | 124 | 174 | 179 | 209 | 221 | 87 | 148 | 155 |
| F2 progeny | As_sdl20 | As-F2-20 | As-F1-C042 | 77 | 80 | 110 | 124 | 174 | 179 | 209 | 210 | 92 | 153 | 152 |
| F2 progeny | As_sdl21 | As-F2-21 | As-F1-C042 | 80 | 84 | 110 | 110 | 176 | 179 | 210 | 222 | 87 | 148 | 151 |
| F2 progeny | As_sdl22 | As-F2-22 | As-F1-C042 | 59 | 77 | 120 | 124 | 179 | 180 | 210 | 222 | 91 | 171 | 154 |
| F2 progeny | As_sdl23 | As-F2-23 | As-F1-C042 | 59 | 82 | 108 | 110 | 178 | 179 | 209 | 221 | 87 | 148 | 155 |
| F2 progeny | As_sdl24 | As-F2-24 | As-F1-C042 | 80 | 93 | 110 | 131 | 174 | 179 | 209 | 224 | 87 | 148 | 153 |
| F2 progeny | As_sdl25 | As-F2-25 | As-F1-C042 | 59 | 80 | 110 | 116 | 176 | 179 | 209 | 217 | 88 | 149 | 151 |
| F2 progeny | As_sdl26 | As-F2-26 | As-F1-C042 | 59 | 77 | 124 | 128 | 178 | 179 | 210 | 213 | 86 | 147 | 151 |
| F2 progeny | As_sdl27 | As-F2-27 | As-F1-C042 | 59 | 77 | 110 | 137 | 178 | 179 | 210 | 217 | 88 | 149 | 152 |
| F2 progeny | As_sdl28 | As-F2-28 | As-F1-C042 | 80 | 84 | 110 | 116 | 179 | 179 | 210 | 222 | 88 | 149 | 153 |
| F2 progeny | As_sdl29 | As-F2-29 | As-F1-C042 | 59 | 59 | 122 | 124 | 179 | 179 | 210 | 217 | 85 | 146 | 154 |
| F2 progeny | As_sdl30 | As-F2-30 | As-F1-C042 | 80 | 84 | 110 | 131 | 178 | 179 | 204 | 209 | 88 | 149 | 151 |
| F2 progeny | As_sdl31 | As-F2-31 | As-F1-C042 | 59 | 84 | 120 | 124 | 176 | 179 | 210 | 210 | 85 | 146 | 152 |
| F2 progeny | As_sdl32 | As-F2-32 | As-F1-C042 | 59 | 93 | 110 | 110 | 179 | 180 | 210 | 224 | 89 | 150 | 153 |
| F2 progeny | As_sdl33 | As-F2-33 | As-F1-C148 | 82 | 84 | 128 | 147 | 178 | 180 | 222 | 228 | 87 | 148 | 155 |
| F2 progeny | As_sdl34 | As-F2-34 | As-F1-C148 | 77 | 84 | 120 | 147 | 176 | 176 | 205 | 222 | 91 | 152 | 154 |
| F2 progeny | As_sdl35 | As-F2-35 | As-F1-C148 | 82 | 93 | 124 | 131 | 176 | 179 | 209 | 221 | 88 | 149 | 152 |
| F2 progeny | As_sdl36 | As-F2-36 | As-F1-C148 | 84 | 93 | 131 | 139 | 176 | 180 | 222 | 224 | 90 | 151 | 151 |
| F2 progeny | As_sdl37 | As-F2-37 | As-F1-C148 | 82 | 84 | 118 | 131 | 176 | 180 | 206 | 222 | 91 | 152 | 153 |
| F2 progeny | As_sdl38 | As-F2-38 | As-F1-C148 | 91 | 93 | 131 | 141 | 174 | 180 | 206 | 222 | 92 | 153 | 151 |
| F2 progeny | As_sdl39 | As-F2-39 | As-F1-C148 | 82 | 84 | 131 | 131 | 180 | 180 | 215 | 222 | 82 | 143 | 151 |
| F2 progeny | As_sdl40 | As-F2-40 | As-F1-C148 | 84 | 91 | 131 | 147 | 176 | 180 | 209 | 222 | 89 | 150 | 155 |
| F2 progeny | As_sdl41 | As-F2-41 | As-F1-C148 | 88 | 93 | 122 | 147 | 180 | 180 | 222 | 222 | 86 | 147 | 152 |
| F2 progeny | As_sdl42 | As-F2-42 | As-F1-C148 | 59 | 93 | 110 | 147 | 176 | 180 | 205 | 209 | 88 | 149 | 154 |
| F2 progeny | As_sdl43 | As-F2-43 | As-F1-C148 | 77 | 93 | 131 | 137 | 176 | 180 | 209 | 222 | 83 | 145 | 151 |
| F2 progeny | As_sdl44 | As-F2-44 | As-F1-C148 | 82 | 84 | 108 | 131 | 178 | 180 | 222 | 228 | 87 | 148 | 155 |
| F2 progeny | As_sdl45 | As-F2-45 | As-F1-C148 | 84 | 93 | 128 | 131 | 174 | 176 | 222 | 222 | 90 | 151 | 151 |
| F2 progeny | As_sdl46 | As-F2-46 | As-F1-C148 | 59 | 93 | 126 | 147 | 176 | 178 | 203 | 209 | 89 | 150 | 154 |
| F2 progeny | As_sdl47 | As-F2-47 | As-F1-C148 | 82 | 84 | 122 | 147 | 176 | 180 | 205 | 222 | 92 | 153 | 152 |
| F2 progeny | As_sdl48 | As-F2-48 | As-F1-C148 | 59 | 84 | 131 | 139 | 176 | 178 | 209 | 217 | 90 | 151 | 155 |
| F2 progeny | As_sdl49 | As-F2-49 | As-F1-C168 | 59 | 77 | 139 | 147 | 176 | 178 | 205 | 219 | 89 | 150 | 154 |
| F2 progeny | As_sdl50 | As-F2-50 | As-F1-C168 | 82 | 93 | 124 | 139 | 176 | 180 | 206 | 219 | 83 | 145 | 153 |
| F2 progeny | As_sdl51 | As-F2-51 | As-F1-C168 | 77 | 93 | 139 | 147 | 178 | 180 | 219 | 232 | 89 | 150 | 154 |
| F2 progeny | As_sdl52 | As-F2-52 | As-F1-C168 | 82 | 93 | 139 | 147 | 176 | 176 | 207 | 219 | 89 | 150 | 153 |
| F2 progeny | As_sdl53 | As-F2-53 | As-F1-C168 | 82 | 93 | 120 | 147 | 176 | 178 | 205 | 222 | 89 | 150 | 154 |
| F2 progeny | As_sdl54 | As-F2-54 | As-F1-C168 | 82 | 93 | 112 | 147 | 178 | 180 | 206 | 222 | 86 | 147 | 153 |
| F2 progeny | As_sdl55 | As-F2-55 | As-F1-C168 | 59 | 77 | 110 | 147 | 171 | 176 | 221 | 222 | 86 | 147 | 153 |
| F2 progeny | As_sdl56 | As-F2-56 | As-F1-C168 | 77 | 77 | 128 | 139 | 176 | 178 | 219 | 222 | 87 | 148 | 154 |
| F2 progeny | As_sdl57 | As-F2-57 | As-F1-C168 | 77 | 91 | 147 | 147 | 174 | 176 | 219 | 224 | 82 | 143 | 151 |
| F2 progeny | As_sdl58 | As-F2-58 | As-F1-C168 | 77 | 82 | 116 | 139 | 176 | 180 | 221 | 222 | 88 | 149 | 155 |
| F2 progeny | As_sdl59 | As-F2-59 | As-F1-C168 | 84 | 93 | 110 | 139 | 176 | 178 | 205 | 219 | 91 | 152 | 152 |
| F2 progeny | As_sdl60 | As-F2-60 | As-F1-C168 | 77 | 93 | 133 | 147 | 176 | 178 | 219 | 226 | 92 | 153 | 152 |
| F2 progeny | As_sdl61 | As-F2-61 | As-F1-C168 | 91 | 93 | 139 | 139 | 176 | 178 | 217 | 219 | 81 | 142 | 152 |
| F2 progeny | As_sdl62 | As-F2-62 | As-F1-C168 | 59 | 77 | 110 | 139 | 176 | 176 | 206 | 219 | 86 | 147 | 152 |
| F2 progeny | As_sdl63 | As-F2-63 | As-F1-C168 | 77 | 93 | 139 | 139 | 178 | 180 | 217 | 222 | 83 | 145 | 153 |
| F2 progeny | As_sdl64 | As-F2-64 | As-F1-C168 | 77 | 80 | 110 | 147 | 174 | 176 | 205 | 222 | 87 | 148 | 153 |
| F2 progeny | As_sdl65 | As-F2-65 | As-F1-C061 | 77 | 84 | 110 | 137 | 174 | 179 | 205 | 206 | 86 | 147 | 153 |
| F2 progeny | As_sdl66 | As-F2-66 | As-F1-C061 | 59 | 84 | 124 | 128 | 175 | 178 | 205 | 222 | 88 | 149 | 153 |
| F2 progeny | As_sdl67 | As-F2-67 | As-F1-C061 | 80 | 82 | 110 | 139 | 175 | 176 | 203 | 209 | 93 | 154 | 153 |
| F2 progeny | As_sdl68 | As-F2-68 | As-F1-C061 | 84 | 84 | 110 | 130 | 175 | 178 | 205 | 219 | 88 | 149 | 153 |
| F2 progeny | As_sdl69 | As-F2-69 | As-F1-C061 | 84 | 88 | 110 | 145 | 175 | 180 | 205 | 217 | 86 | 147 | 152 |
| F2 progeny | As_sdl70 | As-F2-70 | As-F1-C061 | 80 | 80 | 110 | 110 | 175 | 180 | 203 | 206 | 90 | 151 | 152 |
| F2 progeny | As_sdl71 | As-F2-71 | As-F1-C061 | 84 | 95 | 128 | 128 | 175 | 178 | 203 | 205 | 81 | 142 | 152 |
| F2 progeny | As_sdl72 | As-F2-72 | As-F1-C061 | 80 | 91 | 110 | 124 | 174 | 178 | 203 | 205 | 89 | 150 | 153 |
| F2 progeny | As_sdl73 | As-F2-73 | As-F1-C061 | 84 | 97 | 126 | 128 | 175 | 178 | 205 | 205 | 83 | 145 | 152 |
| F2 progeny | As_sdl74 | As-F2-74 | As-F1-C061 | 80 | 82 | 110 | 128 | 175 | 179 | 203 | 206 | 87 | 148 | 152 |
| F2 progeny | As_sdl75 | As-F2-75 | As-F1-C061 | 59 | 80 | 110 | 128 | 175 | 180 | 203 | 206 | 88 | 149 | 151 |
| F2 progeny | As_sdl76 | As-F2-76 | As-F1-C061 | 61 | 80 | 110 | 133 | 175 | 178 | 205 | 222 | 83 | 145 | 157 |
| F2 progeny | As_sdl77 | As-F2-77 | As-F1-C061 | 84 | 84 | 110 | 124 | 175 | 180 | 203 | 215 | 89 | 150 | 152 |
| F2 progeny | As_sdl78 | As-F2-78 | As-F1-C061 | 80 | 84 | 112 | 128 | 174 | 178 | 203 | 206 | 91 | 152 | 154 |
| F2 progeny | As_sdl79 | As-F2-79 | As-F1-C061 | 59 | 80 | 110 | 126 | 174 | 176 | 203 | 205 | 90 | 151 | 151 |
| F2 progeny | As_sdl80 | As-F2-80 | As-F1-C061 | 59 | 80 | 110 | 128 | 174 | 176 | 205 | 236 | 88 | 149 | 154 |
